# Angina Pectoris*Read to Harris County Medical Society, January 14, 1907.

**Published:** 1907-05

**Authors:** Wm. A. Haley

**Affiliations:** Houston, Texas, Prof. Pathology Texas Dental College


For Texas Medical Journal.

### Angina Pectoris.*

BY WM. A. HALEY, M. D., HOUSTON, TEXAS,
Prof. Pathology Texas Dental College.

I shall not discuss the history of this disease only as to its occurrence and relative frequency, as compared to pseudo angina pectoris. Gauthier found that of 71 cases but 3 were true angina, or 4.2 per cent; Gilbert Blanc treated 3835 patients in the St. Thomas Hospital, London, in ten years without finding a single case of true angina. Fraenkle had two distinct cases of true angina in each 250 patients during a year, which seems to be a fairly correct average of cases met with in a general run of practice;[1] while with the pseudo angina it will be met with much more frequently, as nervous persons,—those with bad digestion, etc.,—are liable to neuralgia and neuralgia of the ganglia of the heart, which is the principal feature of pseudo angina, with symptoms sometimes very closely resembling true angina pectoris, yet two distinct diseases, as we must understand them to be.

What we mean by the term "disease": An abnormal performance of certain of the functions of the body. Experience has shown that the signs of disease tend to occur in definite groups. These groups of symptoms come to form distinct ideas in the minds of observers, and each group is called a disease and is given a distinct name. The question then arises, whether we include in the definition of a disease the cause which produces it; whether, for instance, the term diphtheria should be limited to cases of sore throat, in which a certain bacillus is found, or, should be applied to all cases of sore throats? The tendency of the present day is in the direction of defining diseases according to their cause and pathological findings, rather than according to their clinical features.[2]

In dealing with clinical manifestations similar to, yet different, and unaccompanied by the pathological findings, we use a prefix to the name of the disease, where the pathological findings are present to show its similarity and also to indicate its difference, clinically and pathologically.

Now that we have come to a conclusion of the naming of the disease, we will proceed with the etiology of the disease under consideration. It is a disease of adult life, as a rule; more often

---

*Read to Harris County Medical Society, January 14, 1907.

[1]Twentieth Century Practice of Medicine.

[2]Green's Pathology.

found in man than woman. The disposition seems also to be transmitted by inheritance; possibly through the gouty diathesis, syphilis, etc. Other causes may act, such as toxic agencies, but would have a special action on the cell vitality of the part of the anatomy involved in this disease—the coronary arteries, the root of aorta and the myocardium—which is involved in the process of sclerosis in every instance of true angina pectoris. Osler states that without exception the subjects of true angina have arterosclerosis, either general or localized at the root of the aorta, in the coronary arteries or in the myocardium.[4] Gothair and Huchard observed in seventy autopsies changes in the coronaries thirty-eight times, changes in the aorta seventeen times; changes in the myocardium four times. While Wilde found atheroma of the coronaries in every one of six cases which ended in sudden death.[3] It is fair to conclude, considering the other evidence, that as all the seventy cases of Gothair and Huchard had atheromathous changes in the heart and coronaries, except eleven, that this number had some inter-current disease associated with pseudo angina which helped them off, and that all cases of true angina are dependent on arterosclerosis of the heart and associated arteries.

*Clinical Manifestations.*—An attack is generally brought on by excitement, muscular effort, or by exposure to cold. The patient is seized with a pain in the regions of the heart; with a sense of suffocation; the pain extends down to the arm, following the course of the nerves, and up the side of the neck of the left side. The face is pale, and is usually covered with cold sweat; the patient is motionless; the face has an expression of great anxiety and impending death; the pulse is, as a rule, not materially interefered with, except by an increase in tension. The attack usually lasts only a few minutes, after which the patient feels fairly well, in some cases, and in others the patient feels much exhausted for several days after. The sufferer may die in the first or second attack, and in other cases the attacks may extend over a period of years. There is thought to be a condition of transient anemia in the heart muscle associated with spasm when the attack is on, brought about by the extra demand for nutrition of the heart muscle, on account of the muscular or mental effort on the part of the patient and the inability on the part of the associated arteries of the heart to furnish the needed nutrition because of their involvement in sclerosis.

A diagnosis is not easy in many cases, as we have several grades

---

[4]Osler, Practice of Medicine.

[3]Twentieth Century Practice of Medicine.

of the disease and may have the true and the pseudo angina associated in the same patient. From what I have said under clinical manifestations, one would have a pretty good idea of the disease, and would be able to recognize it in most cases. However, it might be well to contrast the symptoms of the two diseases to get a more definite idea:

TRUE ANGINA.

Most common between the ages of 40 and 50 years.

Most common in men. Attacks brought on by exertion.

Attacks rarely periodical or nocturnal.

Not associated with other symptoms.

Vaso-motor form are rare. Agonizing pain and sensation of compression by a vice.

Pain of short duration. Attitude: Silence, immobility.

Lesions: Sclerosis of coronary artery.

Prognosis grave, often fatal.

Arterial medication.

PSEUDO ANGINA.

At every age, even 6 years.

Most common in women. Attacks spontaneous.

Often periodical and nocturnal.

Associated with nervous symptoms.

Vaso-motor form common. Pain less severe, sensation of dissension.

Pain last one or two hours. Agitation and activity.

Neuralgia of nerves of cardioplexus.

Never fatal.

Antineuralgic medication.[5]

Now I think that it is very necessary to make a differential diagnosis, as the pathology of the two diseases is essentially different; one is sclerosis and other neurosis. After coming to a diagnosis it is easy to see that the treatment for one might be decidedly contra-indicated for the other. It would be well to say something with regard to the treatment of both, in order that we might not become confused as to the treatment of either.

In true angina we wouid use alteratives, eliminatives and vasodilators. As alterative, the salts of iodine; vasodilators, the nitrites, eliminatives, alkaline mineral waters and diuretics; while in pseudo angina we would give stimulants, anodynes and recon-

[5]Osler, Practice of Medicine.

structives, such as would be indicated in cases of neurasthenia associated with pain. I shall not enumerate a great list of remedies, as I think it sufficient to mention the class from which they are to be chosen, and allow the physician to choose the drug to be used in each individual case.

It will be seen at once that stimulants would have to be used with great care, and possibly not at all, in true angina; while drugs producing destructive metamorphosis of tissue would not be indicated in such diseases as pseudo angina, where we would only expect results from reconstructive agents.

